# Application of droplet digital PCR in the analysis of genome integration and organization of the transgene in BAC transgenic mice

**DOI:** 10.1038/s41598-018-25001-x

**Published:** 2018-04-27

**Authors:** Ayumi Nakagaki, Asuka Urakawa, Shiori Hirano, Takeru Anami, Tatsuya Kishino

**Affiliations:** 1 Division of Functional Genomics, Center for Frontier Life Sciences, Nagasaki University, Nagasaki, 852-8523 Japan; 2 Nagasaki Prefecture Medical Health Operation Group, Nagasaki, 859-0401 Japan

## Abstract

Transgenic (Tg) mice containing bacterial artificial chromosome (BAC) DNA are widely used for gene expression analysis and gene therapy models because BAC transgenes provide gene expression at physiological levels with the same developmental timing as endogenous genes. To ensure correct interpretation of transgene functions, investigation of the genomic organisation and integration of the BAC transgene is required. Here, we describe a reliable method based on droplet digital PCR (ddPCR) and inverse PCR to estimate copy number, genomic organisation and insertion sites of BAC transgenes in the mouse genome. We generated BAC Tg mice containing fragments of BAC clone RP23-59P20. ddPCR and iPCR analysis showed that the transgene consisted of five fragments of the BAC clone containing the *Mkrn3* gene region, and that the transgene was inserted into *Bckdhb*, homozygous deletion of which causes the maple syrup urine disease phenotype. The ddPCR method described here should prove useful for analysis of genomic organisation and integration of BAC transgenes.

## Introduction

Bacterial artificial chromosomes (BAC) are useful resources for long-range analysis of genomic organisation and gene function. On average, BAC inserts contain 130–200 kb of mouse genomic DNA^1^ and clone numbers and localisations can be found using the University of California, Santa Cruz (UCSC), genome browser (https://genome.ucsc.edu/). Ideally, during BAC transgenesis, BAC DNA introduced by pronuclear injection is integrated as intact BAC molecules into the mouse genome. Transgenic (Tg) mice containing intact BAC molecules are likely to show physiologically-relevant gene expression patterns and copy number-dependent expression levels. BACs are usually used for overexpression studies and rescue experiments of knockout mice; however, these large DNA molecules tend to break prior to and during integration, leading to insertion of fragmented transgenes^2^. Fortunately, mice containing fragmented BAC molecules are also useful to identify long-range gene regulatory elements required for correct tissue-specific or temporal expression^3,4^. In studies using BAC Tg mice, BAC transgene integrity and copy number are not always described in detail. Here, we developed a simple method to investigate the genomic organisation and integration sites of transgenes using a combination of droplet digital PCR (ddPCR) and inverse PCR (iPCR). ddPCR is a newly developed method for performing digital PCR in droplets of water-oil emulsion by microfluidic techniques. It can be adapted for a wide range of biomedical applications due to its digital nature, which provides absolute quantification of nucleic acid target sequences with high sensitivity and accuracy^5^. ddPCR has many potential advantages over real time quantitative PCR (qPCR), such as absolute quantification without the need for standard assays, less influence of inhibitors in PCR, and reproducibility between inter and intra assays. This study introduces the applicability of ddPCR for quantitative detection of copy numbers of transgenes, leading to identification of the integration sites of transgenes by iPCR in BAC transgenic mice.

**Figure 1:**
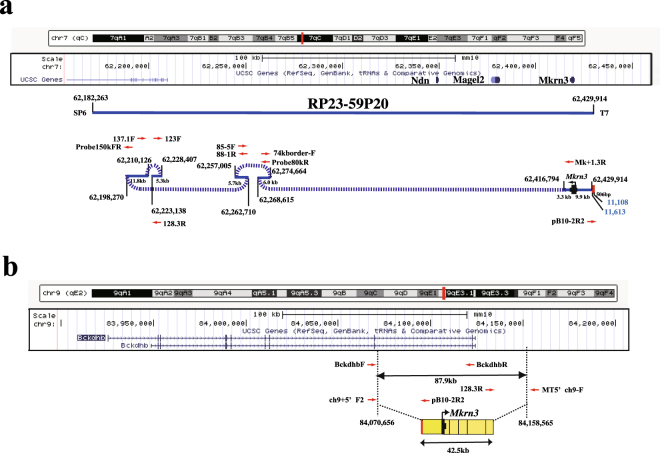
Schematic representation of the genomic organization of BAC clone RP23-59P20 and the transgene fragments of the BAC clone in mouse chromosome 7qC (**a**), and of the insertion site on mouse chromosome 9qE2 (**b**). The upper panels are referenced from the University of California, Santa Cruz, Genome Browser on Mouse Dec. 2011 (GRCm38/mm10). Black numbers indicate the chromosomal positions (chr 7: 62,157,000–62,460,000 and chr 9: 83,899,000–84,240,000), while blue numbers indicate nucleotide positions in cloning vector pBACe3.6 (GenBank accession no. U80929.2). (**a**) In the lower panel, blue lines indicate the original insertion of BAC clone RP23-59P20 and segmented BAC fragments of the transgene. Dotted lines indicate connections between BAC fragments. Red and black boxes indicate fragments originating from the cloning vector and *Mkrn3*, respectively. Red arrows indicate PCR primers used to confirm the joined fragments. (**b**) Instead of the 87.9-kb deletion, including exons 9 and 10 of *Bckdhb*, the 42.5-kb transgene, consisting of five fragments (yellow boxes) of the BAC clone, is inserted into *Bckdhb* intron 8.

Our laboratory uses BAC transgenes to investigate the function of genes in the Prader-Willi syndrome (PWS; [MIM 176270]) critical region. PWS is a neurodevelopmental disorder characterised by neonatal hypotonia, failure to thrive, childhood-onset hyperphagia-associated obesity, intellectual disability, hypogonadism (manifested as genital hypoplasia and incomplete pubertal development) and disturbances of sleep^6,7^. Although PWS is caused by the loss of expression from paternal chromosome 15q11–q13, the molecular mechanisms underlying these clinical features remain poorly understood. The PWS critical region on chromosome 15q11–13, containing *MKRN3*, *MAGEL2, NDN*, *SNRPN* and noncoding SnoRNA clusters (*SNORD115* and *SNORD116*), is largely conserved on chromosome 7 C in mice. Many knockout mice containing a single- or multi-gene deletion in the PWS critical region have been generated, most of which show similar features to the human PWS phenotype, but display incomplete phenotypic spectrums^7,8^. Therefore, to obtain a mouse model with all aspects of the PWS phenotype, a mouse strain with a corresponding loss of all paternal gene expression must be constructed. However, in mice, the loss of all paternally-expressed genes in the critical region results in severe failure to thrive in early life, yielding neonatal lethality^8–10^. To rescue these mice, exogenous gene expression in the PWS critical region is needed, but has not yet been achieved. Therefore, to rescue the neonatal lethality seen in PWS model mice, we attempted to generate Tg mice using BAC clone RP23-59P20, which contains *Ndn*, *Magel2* and *Mkrn3* (Fig. 1). In the process of generating these Tg mice, one of the mouse lines showed neonatal lethality. The genomic organization of the transgene was analysed using the ddPCR and iPCR method, revealing a complicated genomic organisation whereby the transgene was inserted into the branched-chain keto acid dehydrogenase E1 beta polypeptide gene (*Bckdhb*), homozygous mutations in which result in maple syrup urine disease (MSUD).

## Results

### Generation of Tg mice

We generated BAC Tg mice by injecting BAC clone RP23-59P20 into the pronuclei of fertilised C57BL/6 N mouse eggs. Twelve founder mice were genotyped using PCR primers targeting the 5′ and 3′ BAC vector-genomic DNA boundaries (i.e. the SP6 and T7 boundaries). Of these, one line was positive for both the SP6 and T7 boundaries, while another line (10 MT) was only positive for the T7 boundary. These two lines were bred as hemizygous Tg for several generations to ensure that no further segregation of unlinked transgenes occurred. During breeding to generate homozygous Tg mice, line 10 MT demonstrated neonatal lethality in homozygous Tg mice within 48 h of delivery. This line was therefore investigated further.

### Copy number validation of the transgene by ddPCR

**Figure 2:**
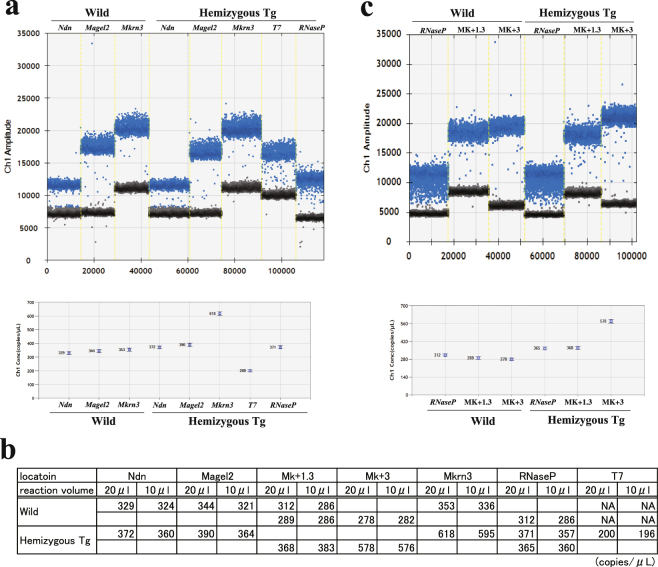
Copy number validation by droplet digital PCR (ddPCR). The upper panels are one-dimensional plots of droplets measured for fluorescence signal (amplitude indicated on *y*-axis) emitted from the genes (**a**) and boundary loci (**c**). Evergreen^™^-bound positive droplets are shown in blue. Negative droplets are shown in black. The lower panels show the concentrations (copies/μl) of the genes and the PCR target sites, as processed by QuantaSoft^™^. The error bars represent the maximum and minimum Poisson distribution for the 95% confidence interval generated by QuantaSoft^™^. (**b**) Comparison of the concentrations generated using 10 and 20 μl of the reaction mixtures for ddPCR analysis.

Because mouse line 10MT was PCR-positive for the T7 boundary but negative for the SP6 boundary, we postulated that fragments of BAC molecules containing the T7 boundary had inserted into the mouse genome. To assess the genomic localisation of the transgene, we employed a ddPCR strategy. We designed PCR primers targeting the *Ndn*, *Magel2* and *Mkrn3* genes as well as the T7 boundary across the BAC. The primers successfully produced a clear distinction between positive and negative partitions in the two-dimensional analysis (Fig. 2a), and the concentrations of each of the genes and the T7 boundary were calculated (Fig. 2b). Concentrations (copies/μl) of *RNaseP*, located on mouse chromosome 19, were used as a control, with a known concentration of two copies per diploid genome in both wild-type and Tg mice. The ddPCR results showed that hemizygous Tg mice had three copies of *Mkrn3* per diploid genome, two copies of *Ndn* and *Magel2*, and one copy of the T7 boundary. In contrast, wild-type mice contained two copies per diploid genome of all of the genes examined and no copies of the T7 boundary containing T7 sequence. We estimated that the three copies of *Mkrn3* per diploid genome detected in hemizygous Tg mice consisted of two endogenous copies and one Tg copy.

Because the ddPCR assay clearly and simply showed the copy number variation within the diploid genomes, primers located between *Magel2* and *Mkrn3* were designed for further ddPCR analysis to identify the candidate region of the boundary, downstream of *Mkrn3* between three copies and two copies per diploid genome. After several rounds of ddPCR analysis, we narrowed the candidate region to a 500-bp segment between the Mk + 1.3 and Mk + 3 loci (Fig. 2b,c). Because the results were reproducible using half volumes (10 μl) of the reaction mixtures (Fig. 2b, Supplementary Fig. S1), half volumes were routinely used for copy number validation in the genome.

### Identification of unknown sequences of the transgene using inverse PCR (iPCR)

**Figure 3:**
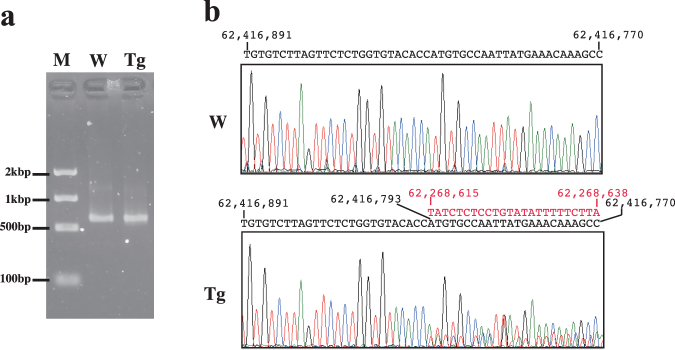
Inverse PCR (iPCR) and sequencing of the boundary region surrounding the Mk + 1.3 locus. (**a**) Agarose gel electrophoresis of nested PCR products stained with ethidium bromide. The gel is the full length gel and lanes have not been cropped or stitched. (**b**) Chromatograms generated from direct sequencing of the iPCR products. Black and red letters above the chromatograms indicate the sequences of chromosomal positions chr 7: 62,416,891–62,416,891 and chr 7: 62,268,615–62,268,638 (on the University of California, Santa Cruz, genome browser (GRCm38/mm10)), respectively. W: wild-type DNA, Tg: hemizygous transgenic DNA, M: molecular size marker.

After narrowing the candidate regions of the boundaries to within several hundred base pairs, iPCR was performed to identify the unknown sequences connected to the known transgene fragments. We individually used restriction endonucleases *Nla*III, *Mse*I and *Sau*3AI, each of which recognizes a 4-bp sequence (CATG, TTAA and GATC, respectively), and digests, on average, every several hundred bases. Digested DNA fragments from wild-type and hemizygous Tg mice were self-ligated and then amplified by nested PCR. To identify the unknown sequence connected to known fragments between the Mk + 3 and Mk + 1.3 loci, the *Sau*3A1-digested fragments were amplified using an inner primer, and then an aliquot of the product from the first PCR assay was amplified using outer primers (Supplementary Fig. S2). Although agarose gel electrophoresis showed that similarly sized products were amplified from wild-type and Tg DNA (Fig. 3a), direct sequencing of the PCR products revealed overlapped sequences of the unknown connected fragment, starting at chr7: 62,268,615 in UCSC Genome Browser on Mouse Dec. 2011 (GRCm38/mm10) (Fig. 3b, Supplementary Fig. S2b), 148 kb centromeric to *Mkrn3* (Fig. 1a).

After confirming the sequence of the joined fragment by PCR using primers Mk + 1.3 R and Probe80KR (Fig. 1a, Supplementary Fig. S3), the neighbouring fragments were further investigated by ddPCR, followed by iPCR. Results showed that the transgene consisted of five fragments (total length 42.5 kb) of the BAC clone, and was inserted in the branched-chain keto acid dehydrogenase E1 beta polypeptide gene (*Bckdhb*) on chromosome 9 (Fig. 1, Supplementary Fig. S3). Instead of insertion of the desired transgene, an 87.9-kb region, including the last two exons of *Bckdhb*, was deleted (Fig. 1b).

### Gene expression in the Tg mice

**Figure 4:**
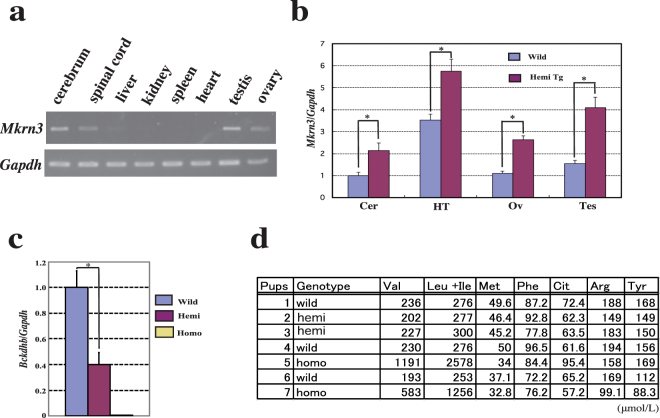
Gene expression and amino acid analysis in the transgenic (Tg) mice. (**a**) Evaluation of *Mkrn3* expression in tissues from adult wild-type mice by real-time PCR (RT-PCR). cDNA concentrations were adjusted relative to the amplification of internal control gene *Gapdh*. Gel pictures were cropped to save space. The full-length gel pictures were shown in the Supplementary Figures (Fig. S4). (**b**) Quantitative RT-PCR analysis of *Mkrn3* expression in tissues from wild-type and hemizygous Tg mice at P9. Error bars indicate the standard error of the means. Asterisks indicate significant differences (p < 0.05). Cer: cerebrum, HT: hypothalamus, Ov: ovary, Tes: testis. (**c**) Quantitative analysis of *Bckdhb* expression in brain tissue. Asterisks indicate significant differences (p < 0.05). (**d**) Amino acid analysis of neonatal blood at P1. Pup #7 was dead prior to blood sampling at P1. Wild: wild type mice, hemi: hemizygous Tg mice, homo: homozygous Tg mice.

Northern blot analysis has previously shown that *Mkrn3* is ubiquitously expressed in almost all tissues in adult mice, with the highest levels found in the testes^11^. Recently, hypothalamic *Mkrn*3 mRNA levels were shown to increase in the arcuate nucleus of both male and female mice during the infantile and early juvenile periods, but decrease between postnatal days 12–15 (P12–15), prior to the onset of puberty^12^. We first analysed *Mkrn3* expression in adult tissues using RT-PCR. Expression was detected in the cerebrum, spinal cord, ovaries and testes, but was not observed in the liver, kidney, spleen or heart of wild-type mice (Fig. 4a). To see if transgenic *Mkrn3* expression is regulated by imprinting mechanism, because endogenous *Mkrn3* is imprinted in various tissues, where it is expressed exclusively from the paternal chromosome, *Mkrn3* expression analysis was performed by qPCR, on neonatal brains of Tg mice inheriting the transgene either maternally or/and paternally. Regardless of maternally or paternally inheritance of the transgene, total expression level of *Mkrn3* in hemizygous Tg mice was 1.8~2.3-fold higher in the neonatal brain, while that in homozygous Tg mice was 2.7-fold higher than in the wild-type brain (Supplementary Fig. S5). *Mkrn3* expression in various tissues in hemizygous Tg mice was analysed to see if the transgenic *Mkrn3* gene was expressed with the same tissue specificity as the endogenous *Mkrn3* gene. qPCR analysis revealed that total expression levels were approximately 2-fold higher in all tissues examined than levels observed in wild-type mice (Fig. 4b).

As transgenic insertion in the 10MT mouse line resulted in deletion of an 87.9-kb region containing the last two exons of *Bckdhb*, we examined *Bckdhb* expression in the Tg mice. RT-PCR analysis showed that *Bckdhb* expression in the brain was decreased by almost 50% in hemizygous Tg mice, and no expression was observed in homozygous Tg mice (Fig. 4c). Because *Bckdhb* is involved in MSUD, a metabolic disorder affecting branched-chain amino acids, and homozygous Tg mice died within 48 h of delivery, we analysed the levels of branched-chain amino acids in neonatal blood samples (Fig. 4d). At P1, valine and leucine + isoleucine levels were nearly 5- and 9-fold higher, respectively, in homozygous Tg pups than in heterozygous and wild-type pups.

## Discussion

In Tg mouse studies, analysis of copy number and integration sites of the transgenes are essential for downstream analysis of gene expression. In most cases, Southern blot analysis and quantitative PCR (qPCR) assays are used for copy number validation. Southern blotting is used for large-scale analysis of transgene structure, while qPCR assays, which can measure 1.25- to 1.5-fold differences in gene expression under ideal conditions, can be useful for evaluating copy number variations associated with gene/chromosomal dosage^13^. However, it is often difficult and laborious to establish ideal conditions with appropriate calibration curves for qPCR assays. Therefore, in this study, we used ddPCR in place of a qPCR approach. The ddPCR system partitions nucleic acid samples into thousands of nanolitre-sized droplets of water-oil emulsion. PCR amplification is carried out within each droplet, and the fraction of PCR-positive droplets is analysed using Poisson statistics to determine the target DNA template concentration in the sample. In ddPCR, the counting of the PCR-positive droplets is definitive and does not require the ideal conditions that are essential for qPCR, meaning that ddPCR results are more reproducible than those obtained by qPCR analysis. In addition, when compared with qPCR, ddPCR can accurately measure smaller fold differences in gene expression (<1.2-fold)^14^.

In the analysis of genomes modified with transgenes made up of fragmented BAC molecules, copy number evaluation at multiple loci across the BAC provides information about the transgene structure on a larger scale. Consistent copy numbers of more than three copies per diploid genome of all loci across the BAC indicates the genome contains one or more intact BAC molecules, while variable copy numbers between several loci across the BAC suggests that the genome contains fragmented BAC molecules. To compare copy numbers of multiple loci per diploid genome, the ddPCR method is a convenient and economical option because the QX200™ Droplet Generator can treat eight samples at a time using DG8™ cartridges, and half volumes of the reaction mixtures is enough for simple comparison of copy numbers between each sample (Fig. 2b). Tandem insertion of BAC fragments sometimes occurs in the mouse genome. To separate tandem gene copies and accurately determine copy numbers, DNA fragmentation by restriction digestion prior to droplet generation is recommended by the manufacturer. Four-base cutters that do not cut either the target or reference amplicon are preferred. In the present study, we did not perform DNA fragmentation by restriction digestion prior to droplet generation. However, when ddPCR results show ambiguous copy numbers per diploid genome, or copy numbers that are not whole numbers (e.g. copy numbers from 2.4 to 2.6 per diploid genome), DNA fragmentation should be carried out to confirm the tandem insertion of the BAC fragments, or to rule out the possibility of mosaicism of the transgenes.

In this study, we first focused on the *Mkrn3* gene region, and could identify the candidate region of the border of the fragment containing *Mkrn3* to within several hundred base pairs. To identify the sequence of the unknown neighbour fragment connected to the known fragment containing *Mkrn3*, we used an iPCR method, which involves a series of restriction digests and self-ligation steps, resulting in a looped fragment that can be primed for nested PCR from the known sequence. PCR-based chromosome walking techniques, including iPCR, are the main methods used to identify transgene-flanking sequences. The success of the PCR amplification in these techniques depends on the distance between the restriction digestion sites and the known region, meaning that the digested DNA fragments are sometimes too large for standard PCR amplification. Narrowing the candidate regions of the borders by ddPCR made it possible to select suitable four-base-cutter restriction endonucleases that digest, on average, every several hundred base pairs, leading to successful amplification by iPCR.

Finally, we confirmed that the transgene consists of five fragments of the BAC clone, with a total length of 42.5 kb, and was inserted into the *Bckdhb* gene located on chromosome 9. It is not clear whether the observed complicated genomic organization of the transgene is specific to the RP-23 59P20 BAC clone. We could not find any previous descriptions of complicated genomic organization of BAC transgenes made up of fragmented BAC molecules. A previous report described the generation of BAC Tg mice containing a transgene consisting of the *Mkrn3*, *Magel2* and *Ndn* genes to rescue PWS model mice; however, the genomic organization and integration of the BAC transgene was not described^15^. The BAC clone was mapped to the large imprinted domain, where imprinted expression of the genes is regulated by a bipartite *cis*-acting imprinting centre (IC) located ~2.4 Mb downstream of *Mkrn3*. The long-range interactions between the IC and the imprinted genes in the domain are thought to form a chromatin holocomplex structure^16^ similar to an active chromatin hub^17,18^. Such complexity in the chromatin conformation may affect the fragmentation and integration of the BAC molecule in the mouse genome.

Here we described a transgene with a complicated structure, but did not rule out the possibility of additional transgenes with small fragments sizes, where copy numbers were not analysed at loci across the BAC by ddPCR. Recently, successful use of a high-throughput next-generation sequencing (NGS) platform to characterise transgene integration has been reported^19^. The application of NGS to the characterisation of Tg animals will be significant but laborious, and will be a very expensive option. However, it may prove useful in cases where traditional PCR-based methods fail to provide enough information about the transgenes.

*MKRN3/ZNF127* is one of the imprinted human genes identified locationally in the imprinted domain of the PWS critical region^20^. The mouse orthologue, *Mkrn3/Znf127*, is also imprinted in various mouse tissues, where it is expressed exclusively from the paternal chromosome^11^. *Mkrn3* encodes a RING zinc-finger protein of the Makorin family, which presumptively possesses ubiquitin-protein ligase (E3) activity^21^, and may play a role in the imprinted phenotype of mouse models of PWS. However, since 2013, *MKRN3* mutations have also been reported in families with central precocious puberty^12,21,22^. This suggests that the function of *MKRN3* is relevant to pubertal initiation, although PWS patients usually have delayed or incomplete puberty despite the deletion of *MKRN3*.

In the process of generating BAC RP23-59P20 transgenic mice, the *Mkrn3* Tg mouse line was produced. The transgene includes 9 kb of the upstream region and 6 kb of the downstream region of *Mkrn3*, as well as the major *Mkrn3* transcript (~2.9 kb)^11^. Regardless of maternally or paternally inheritance of the transgene, the level of *Mkrn3* expression from the transgene was almost equal to that from the endogenous gene. Such physiologically relevant gene expression from the transgene contrasts with results observed in modified BAC Tg mice containing a *Ndn-eGFP* BAC transgene, in which the *Ndn* coding sequence was replaced with an *eGFP* sequence under the control of the *Ndn* promoter^23^. The *Ndn-eGFP* BAC transgene showed transcriptional regulation equivalent to monoallelic expression of *Ndn*, suggesting the possibility of promoter competition for transcriptional activators, or a mechanism involving physical contact in *trans* between promoters. The original BAC clone used in the *Ndn* Tg mouse study, BAC*603M20* (referred as BAC109)^24^, contains a 103-kb *Not*I insert comprising the *Ndn* and *Magel2* genes. In comparison, BAC clone RP23-59P20 contains a 248-kb *Eco*RI insert, including the 103-kb *Not*I insert, which is completely deleted in our fragmented transgene. It remains unclear as to whether, in the presence of the *Ndn* promoter, *Mkrn3* is independently expressed from the transgene, or whether it is transcriptionally affected by the *Ndn* promoter in the transgene. A chromatin holocomplex structure between the IC and the promoters of imprinted genes, especially *Mkrn3* and *Ndn*, has been suggested^16^.

To determine whether Tg *Mkrn3* expression is physiologically relevant to the endogenous expression, *Mkrn3* expression patterns in adult tissues were analysed by RT-PCR. Although previous northern blot analysis showed ubiquitous expression of *Mkrn3*^11^, in the current study, *Mkrn3* expression was detected only in neural and gonadal tissues by RT-PCR. These discrepancies between northern blot and RT-PCR analysis results may be caused by the large size of the RT-PCR product (764 bp), which would be difficult to amplify from *Mkrn3* cDNA because the 3′ UTR of *Mkrn3* is AU-rich, characterized by AUUUA elements, which are suggested to be unstable^10^. Despite this possible RNA instability, high levels of *Mkrn3* expression in hypothalamus and gonadal tissues provide evidence that Mkrn3 is involved in the regulation of gonadal function at both the hypothalamic-pituitary level and the gonadal level in the hypothalamic-pituitary-gonadal axis. Mice lacking an active paternal allele of *Mkrn3* have previously been described as normal in appearance^11^, and the Tg mice generated in the current study, with demonstrated overexpression of *Mkrn3*, are fertile without any pubertal abnormalities. However, careful investigation of phenotype, especially pubertal timing, will be carried out.

Insertion of the transgene in the current model resulted in the destruction of *Bckdhb*, which encodes the beta subunit of the BCKDH E1 component. Homozygous mutations of the human orthologue, *BCKDHB*, cause MSUD [MIM 248600], an inborn metabolic disease with an autosomal recessive pattern of inheritance. *BCKDHB* is one of four genes that control the formation of the BCKDH enzyme complex. Based on residual BCKDH complex activity, clinical presentation and onset age, MSUD can be divided into five forms^25^. The classic form, with less than 2% residual activity, is the most severe type, and accounts for almost 75% of cases. The classic form of MSUD (cMSUD) is mostly caused by mutations in *BCKDHA*, *BCKDHB* and *DBT*. To date, at least 93 mutations in *BCKDHB* have been described in MSUD patients, according to the Human Gene Mutation Database (www.hgmd.cf.ac.uk), most of which are missense mutations. Mice with targeted deletions of *Dbt*, which encodes the E2 subunit of BCKDH, have been used as a cMSUD model, with a phenotype mimicking that of patients with cMSUD. The present BAC Tg mice also show neonatal lethality and have markedly elevated levels of branched-chain amino acids, indicating that they may be used as another cMSUD model.

In summary, we developed a quick and reliable method for estimating copy number and integrity of BAC transgenes in a genome using a combination of ddPCR and iPCR. In BAC Tg mice containing fragments of BAC clone RP23-59P20, the transgene consisted of five fragments containing the *Mkrn3* region, and was inserted into *Bckdhb*. Expression analysis revealed that Tg *Mkrn3* expression levels are physiologically relevant, and that *Bckdhb* is not expressed in homozygous Tg mice, resulting in another cMSUD mouse model.

## Materials and Methods

### Generation of BAC Tg mice

All experiments, designed to minimise animal pain, were carried out with the approval of the Nagasaki University Institutional Animal Care and Use Committee. BAC Tg mice were generated by pronuclear injection of fertilised C57BL6/N eggs containing BAC DNA. BAC DNA (clone RP23-59P20) was diluted to 5 µg/ml in phosphate buffered saline, and then picolitre volumes of BAC DNA (5 µg/ml) in circular form were microinjected into the male pronucleus of fertilised oocytes^26^. Litters were screened for BAC integration into somatic cells by genotypic selection as described below, using DNA isolated from tail clips taken at the time of weaning^27^. Germ line transmission was established by mating tail-positive progeny with wild-type congenic mice. Detailed information on the insert in BAC clone RP23-59P20 is available from the UCSC genome browser (http://genome.ucsc.edu/index.html). Tg mice were maintained in their original genetic background (C57BL6/N), and the transgene was transmitted to the offspring from a hemizygous transgenic parent. Wild-type and hemizygous Tg littermates across multiple litters were used for the experiments. Homozygous Tg mice were generated by crossing of hemizygous littermates, but not maintained because of neonatal lethality.

### Genotypic selection and PCR assays

Genotyping of the BAC Tg mice was performed by PCR analysis. PCR assays were designed using primers targeting the T7 and SP6 sequences in BAC vector pBACe3.6, and primers 59P20T7qR and 59P20SP6qR, which were specific to the 5′ and 3′ boundaries of mouse genomic DNA within the BAC clone. All primers used for PCR, qPCR and ddPCR analysis are listed in Supplementary Table 1.

### Copy number analysis of the transgene using ddPCR

Genomic DNA extracted from the wild-type and BAC Tg mice was diluted with distilled water to 10 µg/ml and then used for all ddPCR experiments to normalise the DNA concentration between samples. As recommended for ddPCR, primers were designed to amplify products of 100–200 bp. The reaction mixtures contained ddPCR EvaGreen Supermix (Bio-Rad Laboratories, Hercules, CA, USA), primers (final concentration, 100 nM; Supplementary Table 1) and template DNA (2 μl) in a final volume of 20 μl. Each reaction was then loaded into a sample well of an eight-well disposable cartridge (DG8™; Bio-Rad Laboratories) along with 70 μl of droplet generation oil (Bio-Rad Laboratories). Droplets were formed using a QX200™ Droplet Generator (Bio-Rad Laboratories) as per the manufacturer’s instructions. Droplets were then transferred to a 24-well PCR plate, heat-sealed with foil and amplified to the end point using a conventional thermal cycler (95 °C for 5 min, followed by 40 cycles of 94 °C for 30 s and 60 °C for 1 min, and final extension at 98 °C for 10 min). The annealing temperature and cycle number were optimised for incremental separation between positive and negative partitions in ddPCR. The resulting products were scanned on a QX200 Droplet Reader (Bio-Rad Laboratories), and the data analysed using QuantaSoft^™^ software (Bio-Rad Laboratories). When a half volume (10 μl) of the reaction mixture was applied, 40 μl of droplet generation oil was used to form droplets.

### iPCR and sequencing

Extracted genomic DNA was also digested with *Sau*3AI, *Mse*I or *Nla*III at 37 °C for 2 h. The digested DNA was then heat-inactivated at 65 °C for 20 min, and circularized by self-ligation with an equal volume of Instant Sticky-end Ligase Master Mix (New England Biolabs, Beverly, MA, USA). Nested amplification around the circular DNA using primers oriented in a direction opposite to that of conventional PCR was performed using reaction mixtures containing DNA template, 1 × Ex Taq buffer (containing 2.0 mM of MgCl_2_), 250 μM of dNTP mix, 0.5 μM of each primer, and 0.25 U/μl of Ex Taq DNA Polymerase (Takara Bio, Otsu, Japan). Thermal cycler parameters used in the first round of amplification were as follows: 94 °C for 6 min, followed by 35 cycles of 94 °C for 30 s, 55 °C for 30 s and 72 °C for 2 min, and a final extension at 72 °C for 10 min. One microlitre of product from the first round of PCR was then used as template for a second round of amplification using the same cycling conditions. PCR amplifications were performed in an ABI9700 Thermal Cycler (Applied Biosystems, Foster City, CA, USA), and PCR products were sequenced using an ABI3130 Sequencer (Applied Biosystems).

### cDNA synthesis

Total RNA was isolated from tissues using an RNeasy Mini Kit (QIAGEN, Valencia, CA, USA) according to the manufacturer’s protocol. The RNA was treated with amplification-grade DNaseI (Invitrogen, Grand Island, NY, USA) to degrade any genomic DNA present in the sample. cDNA was then generated from total RNA using Superscript III RNaseH Reverse Transcriptase (Invitrogen) primed with random hexamer primers. First-strand cDNA was synthesised at 42 °C for 50 min. mRNA-cDNA chains were denatured and the reverse transcriptase activity was arrested by incubating the mixtures at 70 °C for 15 min. Duplicate reactions minus the reverse transcriptase were included as negative controls.

### Quantitative analysis of gene expression by RT-PCR

The cDNA was used as template for RT-PCR assays to quantify the expression of *Mkrn3* and *Bckdhb* in the wild-type and Tg mice. Assays were carried out using SYBR Premix Ex Taq (Takara Bio) and an ABI Prism 7900 RT-PCR system (Applied Biosystems). Each sample was analysed in at least triplicate to control for PCR variation. To standardise each experiment, the results were represented as a percentage of expression, calculated by dividing the average value of the expression of the target gene by that of an internal control gene, *Gapdh*. Each experiment was repeated two or three times using independent RNA samples. All data were presented as the mean ± standard deviation of the means. The p*-*values were calculated using a two-tailed *t*-test.

### Amino acid analysis

Blood collected from the seven littermates delivered from the hemizygous female mouse mated with the hemizygous male mouse at P1 was spotted onto filter paper (ToyoRoshi Kaisha Ltd., Tokyo, Japan) routinely used for blood amino acid analysis during newborn screening. The presence of branched-chain amino acids (leucine, isoleucine and valine) was determined by tandem mass spectrometry using an API3200 apparatus (AB Sciex, Foster City, CA, USA) as per the manufacturer’s instructions. MS^2^ Screening Neo Siemens (Siemens Healthineers, Erlangen, Germany) was used as the quality control material.

## Electronic supplementary material

supplementary information
