# Supplementary material for: Application of droplet digital PCR in the analysis of genome integration and organization of the transgene in BAC transgenic mice
Source: Sci Rep. 2018 Apr 27;8:6638. doi: 10.1038/s41598-018-25001-x (PMC5923295; doi:10.1038/s41598-018-25001-x)
Supplement: Supplementary file 1 — supplementary information [file 41598_2018_25001_MOESM1_ESM.docx]

**Application of droplet digital PCR in the analysis of genome integration and organization of the transgene in BAC transgenic mice**

Ayumi Nakagaki^1^, Asuka Urakawa^1^, Shiori Hirano^1^, Takeru Anami^2^, Tatsuya Kishino^1^

^1^Division of Functional Genomics, Center for Frontier Life Sciences, Nagasaki University, Nagasaki, Japan, 852-8523

^2^Nagasaki Prefecture Medical Health Operation Group, Nagasaki, Japan, 859-0401

SUPPLEMENTARY INFORMATION

Supplementary Fig. S1–S5, with legends

Supplementary Table. 1

**
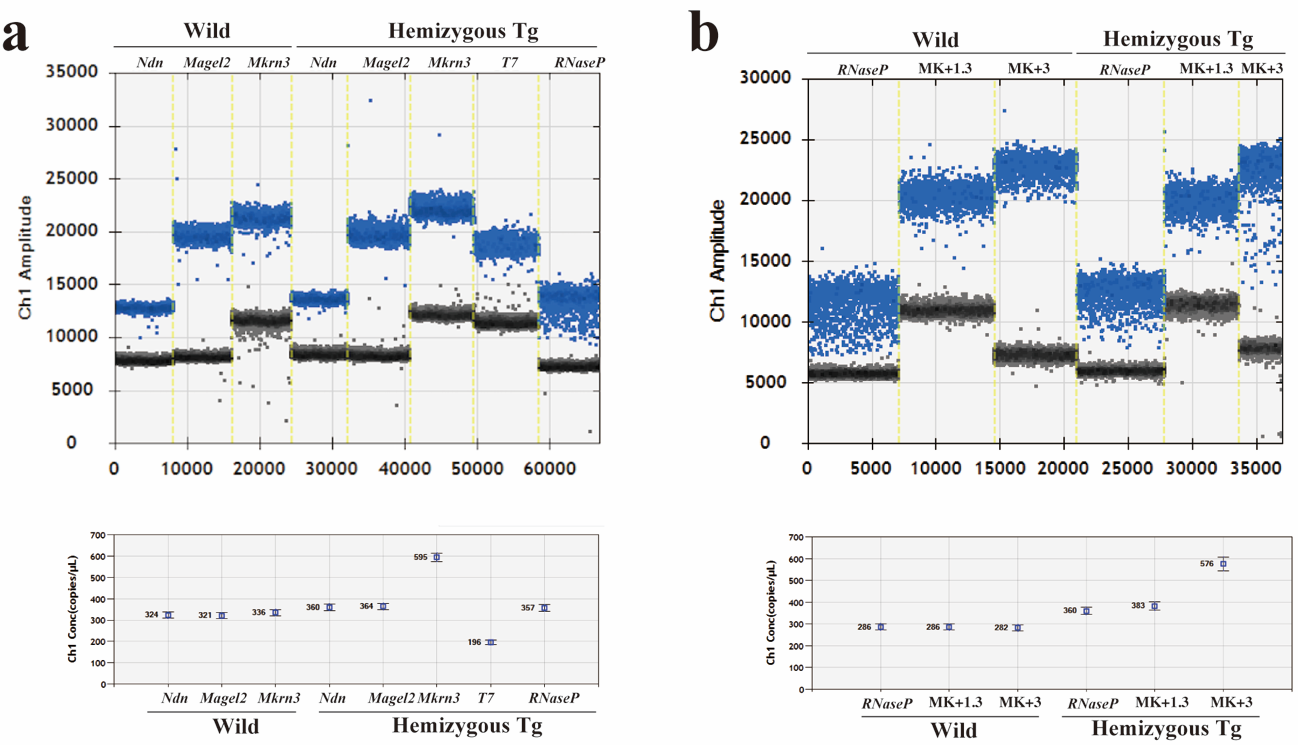
**

**Supplementary Fig. S1** Copy number validation by droplet digital PCR using half volumes (10 μl) of the reaction mixtures. The upper panels are one-dimensional plots of droplets measured for fluorescence signal (amplitude indicated on y-axis) emitted from the genes (a) and boundary loci (b). Evergreen^™^-bound positive droplets are shown in blue. Negative droplets are shown in black. The lower panels show the concentrations (copies/μl) of the genes and PCR target sites as processed in QuantaSoft^™^. The error bars represent the maximum and minimum Poisson distribution for the 95% confidence interval generated by QuantaSoft^™^.

**
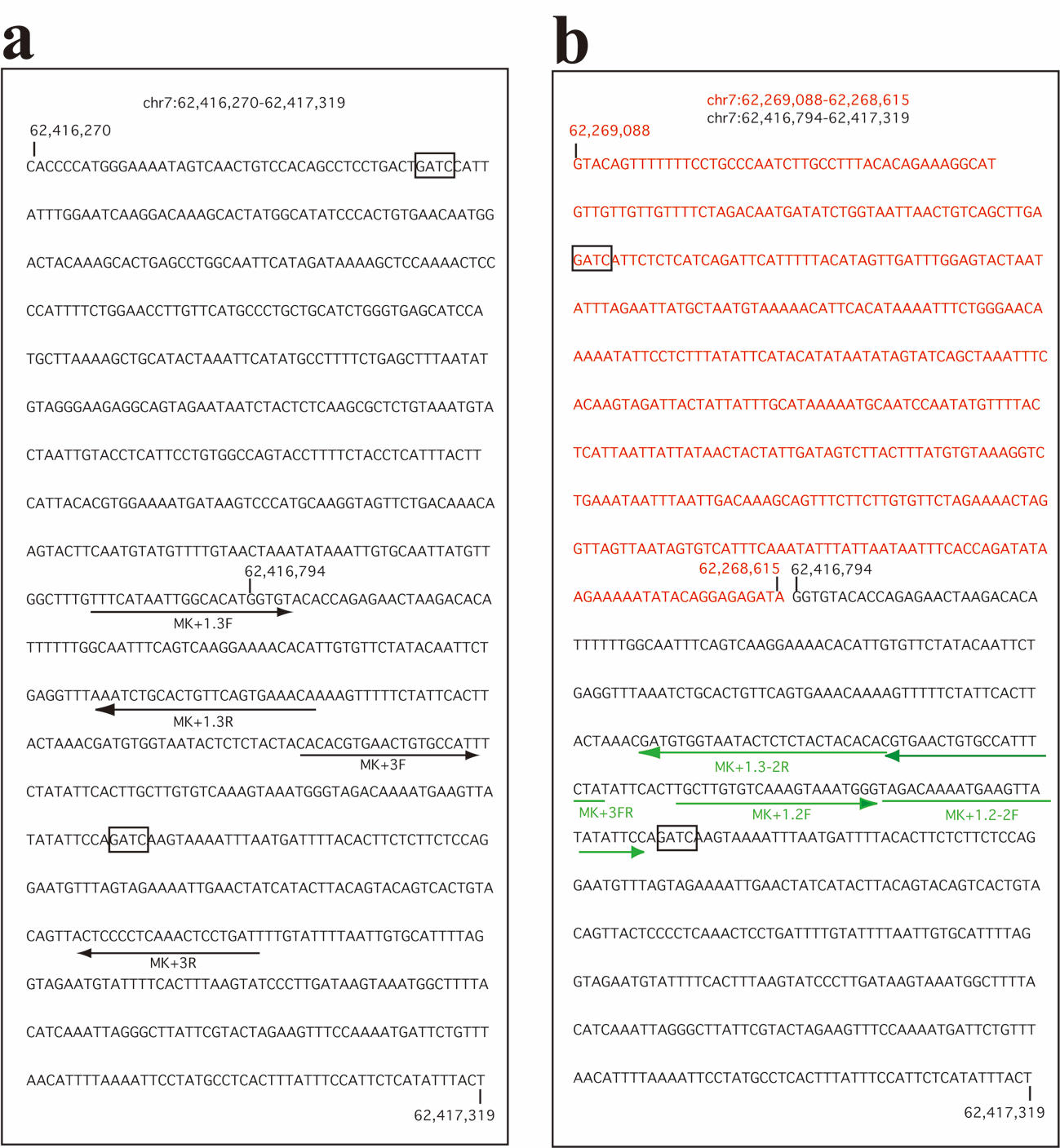
**

**Supplementary Fig. S2** Sequences of the boundary region around the Mk+1.3 locus in wild-type and transgenic mice. (a) Wild-type sequence corresponding to chromosomal position chr 7: 62,416,270–62,417,319 in University of California, Santa Cruz, Genome Browser on Mouse Dec. 2011 (GRCm38/mm10)). (b) Transgene sequence around the boundary. The sequence corresponding to chromosomal position chr 7: 62,269,088–62,268,615 (red letters) is joined to the sequence corresponding to chromosomal position chr 7: 62,416,794–62,417,319 (black letters). Primers used for droplet digital PCR and inverse PCR (iPCR) are shown as black and green arrows, respectively. *Sau*3A1 digestion followed by self-ligation was performed to identify the unknown joined sequence by iPCR. *Sau*3A1 recognition sites (GATC) are framed.


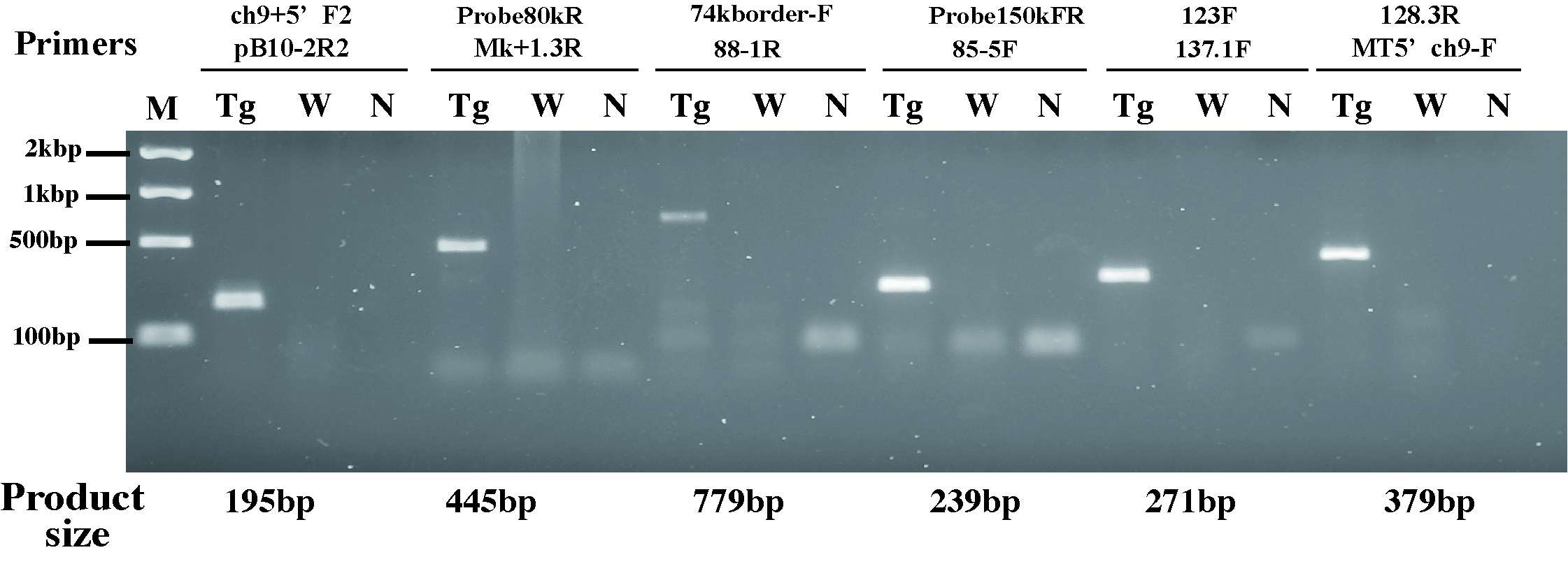


**Supplementary Fig. S3** Agarose gel electrophoresis of PCR products. Joined fragments of the transgene were detected by PCR using the boundary primers (Fig. 1A, Supplementary Table 1). The gel is the full length gel and lanes have not been cropped or stitched. Tg: hemizygous transgenic DNA, W: wild-type DNA, N: dH_2_O, M: molecular size marker.


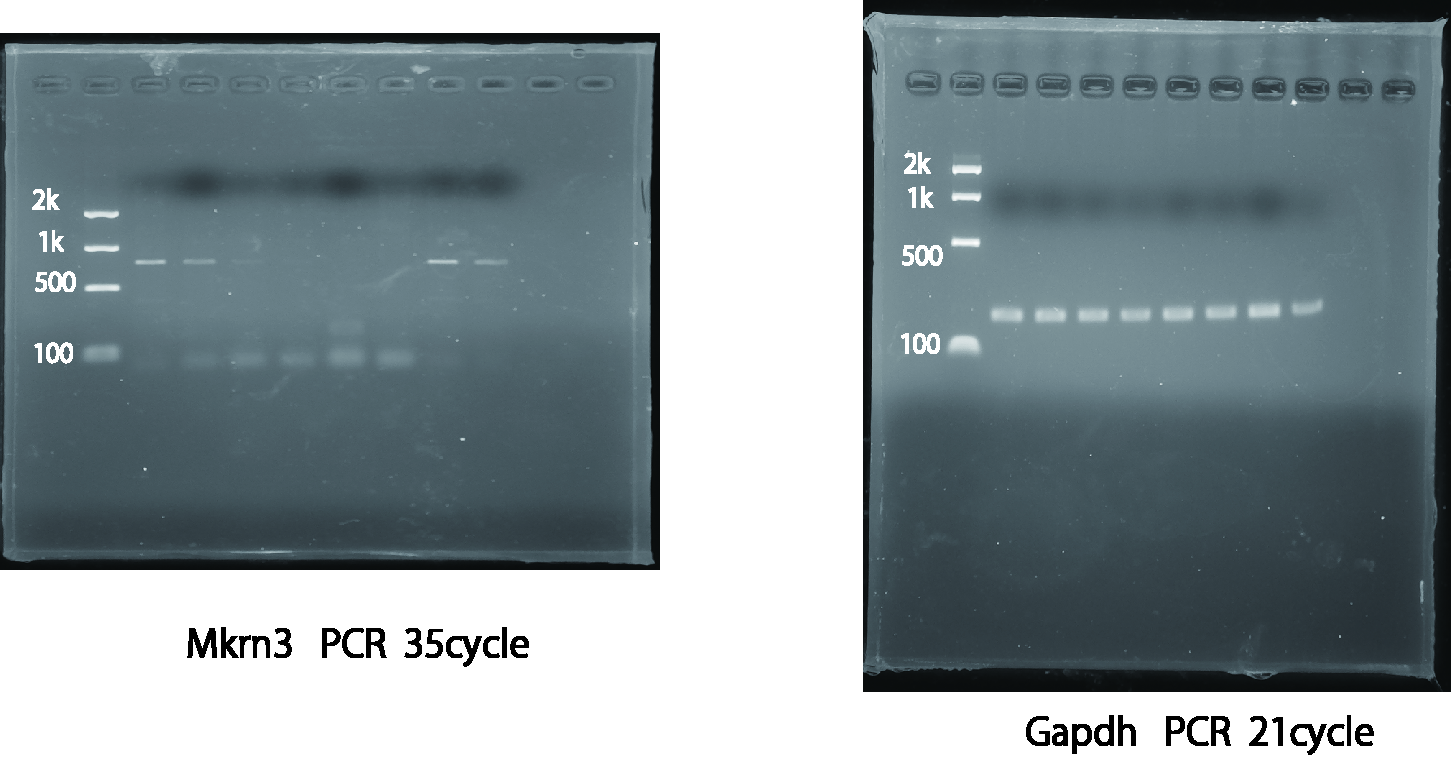


**Supplementary Fig. S4** Full pictures of the gels in Figure 4a


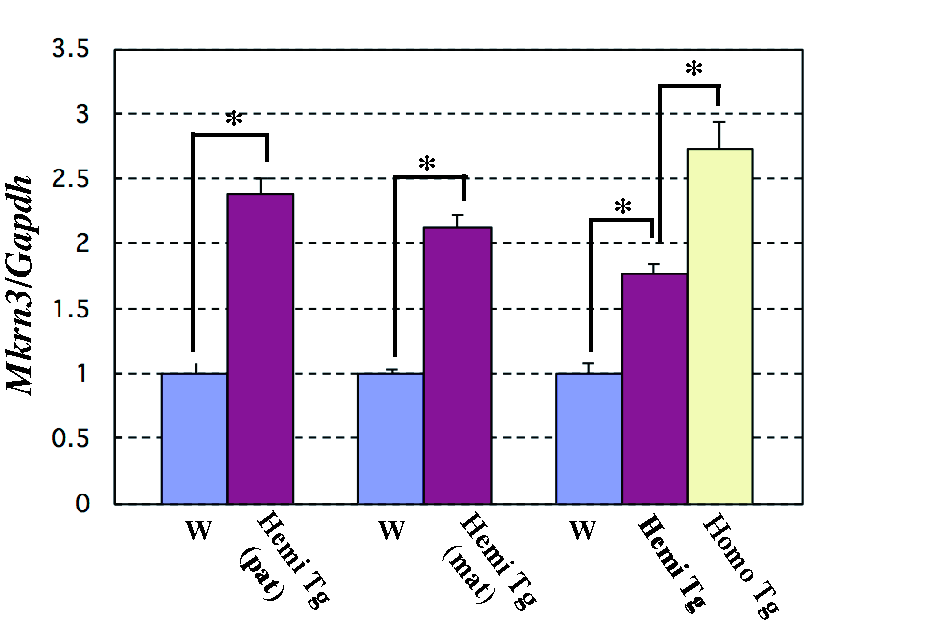


**Supplementary Fig. S5** Quantitative RT-PCR analysis of *Mkrn3* expression in neonatal brains from wild-type (W), hemizygous (Hemi) Tg inheriting the transgene either maternally (mat) or paternally (pat) and homozygous(Homo) Tg mice. Error bars indicate the standard error of the means. Asterisks indicate significant differences (p<0.05).

**Supplementary Table. 1**
